# One-year outcome trends in Japanese patients with rosacea: insights from a real-world study

**DOI:** 10.1093/skinhd/vzaf124

**Published:** 2026-02-06

**Authors:** Yoshimasa Nobeyama, Yoshiko Aihara

**Affiliations:** Department of Dermatology, The Jikei University School of Medicine, Tokyo, Japan; Ai Dermatology Clinic, Utsunomiya, Japan

## Abstract

**Background:**

Rosacea is a chronic inflammatory disorder that primarily affects the facial region, significantly impacting quality of life (QOL). Outcomes in patients with rosacea may vary significantly depending on ethnicity, climatic conditions, healthcare system and cultural background. After unifying these factors, outcomes in rosacea should be evaluated over the long term.

**Objectives:**

To examine long-term outcomes in Japanese patients with rosacea using data obtained from a single institution.

**Methods:**

In this retrospective observational study, data from 63 Japanese patients with rosacea, including 42 patients who were followed up over 1 year, were analysed. Patients were treated with topical agents, with or without oral minocycline, and advised to avoid common triggers such as sun exposure and skin dryness. These data were evaluated using the Dermatology Life Quality Index (DLQI), visual analogue scale (VAS) and Investigator’s Global Assessment (IGA) scoring systems.

**Results:**

Baseline data for the 63 patients showed that QOL was commonly impaired by uncomfortable sensations, such as itch, as well as by disfigurement. Most patients considered rosacea to have a small-to-moderate impact on their lives. DLQI scores were significantly correlated with VAS scores for itch, burning sensation, flushing and hypersensitivity. IGA scores were significantly correlated with VAS scores for flushing and with DLQI scores. Follow-up data for 42 patients over 1 year showed that VAS scores for itch, burning sensation, flushing and hypersensitivity, as well as DLQI and IGA scores, were significantly decreased over time. Topical therapies did not affect the scores throughout the clinical course.

**Conclusions:**

This study was conducted at a single institution in a specific geographical and climatic setting for Japanese patients with rosacea. These findings may inform follow-up care for Japanese patients and contribute to the exploration of ethnic and geographical variations in the presentation and progression of rosacea.

## Teaser

This study was conducted at a single institution within a specific geographic and climatic setting to evaluate long-term trends in rosacea outcomes among Japanese patients. One-year follow-up data initially revealed (i) a significant, time-dependent improvement in both subjective symptoms affecting quality of life and objective findings and (ii) sex differences in the clinical course of certain subjective symptoms. These findings may help guide follow-up care for Japanese patients and contribute to understanding ethnic variations in rosacea.

What is already known about this topic?

- Short-term outcomes in Japanese patients with rosacea have already been reported.
- In addition, long-term outcomes in a relatively heterogeneous group of patients with rosacea have also been analysed.

What does this study add?

- This study provides additional insights into the long-term outcomes of Japanese patients with rosacea, whose unique backgrounds encompass climatic conditions, ethnic background, cultural factors and the healthcare system.
- These insights may inform follow-up care for Japanese patients and facilitate exploration of ethnic and geographical variations in the presentation and progression of rosacea.

Rosacea is a chronic inflammatory disorder primarily affecting the facial skin, with prominent involvement of the cheeks, nose, chin, forehead and periorbital area.^1^ Cutaneous manifestations include persistent facial erythema, phymas, papules, pustules, telangiectasia and flushing. Rosacea is known to significantly impair the quality of life (QOL) of patients.^2^

Several reports have documented favourable outcomes associated with QOL by therapeutic interventions in clinical trials for various treatment options, such as metronidazole gel (0.75%),^3^ sodium sulfacetamide 10%/sulfur 5% emollient foam,^4^ doxycycline or minocycline,^5^ pulsed dye laser,^6,7^ pulsed dye laser and/or neodymium-doped yttrium aluminium garnet laser,^8^ noninsulated fractional microneedle radiofrequency,^9^ botulinum toxin A^10^ and decorative cosmetics.^11^ However, real-world data on outcomes focusing on the long-term clinical course remain limited, despite the need for such data given the chronic nature of rosacea.

Development of rosacea can vary depending on genetic background, including ethnic factors, as well as geographical, climatic and meteorological factors. Recent genome-wide association studies have identified genomic regions, including human leucocyte antigen-coding regions, that are linked to the development of rosacea.^12,13^ Furthermore, several epidemiological studies have suggested that rosacea in individuals with skin of colour is more prevalent in women than in men and is commonly characterized by papules and pustules, as well as by associations with prior steroid use, *Demodex* infestation and sun exposure.^14–16^ In addition, a recent study suggests that Japanese patients with rosacea, whose Fitzpatrick skin phototypes are predominantly III and IV, experienced worsening symptoms due to airborne exposure to seasonal pollen,^17^ which varies in type and amount according to geographical and climatic conditions.

Besides genetic factors and geographical, climatic and meteorological influences, rosacea outcomes can also be affected by cultural background and healthcare systems. Considering these variations, outcomes in rosacea should be evaluated throughout the year for a single ethnic group with a homogeneous cultural background in a specific geographical area. Therefore, this study aimed to investigate (i) the long-term outcomes and (ii) the relationship between subjective symptoms and objective findings before treatment initiation in Japanese patients with rosacea, using data collected from a single institution under consistent climatic conditions.

## Patients and methods

### Patients

This retrospective observational study was approved by the Ethics Committee of The Jikei University School of Medicine. Informed consent was obtained via an opt-out form on the website. The study included Japanese patients with rosacea residing in Tochigi Prefecture, Japan, who met the following criteria: (i) referrals to our clinic in Utsunomiya, Tochigi Prefecture, Japan, from February 2023 to January 2024, and (ii) fulfillment of the diagnostic criteria for rosacea according to the ROSacea COnsensus (ROSCO) panel.^18^ Other inflammatory facial diseases, such as seborrhoeic dermatitis, contact dermatitis, lupus erythematosus, lupus miliaris disseminatus faciei, eosinophilic pustular folliculitis, photosensitive dermatopathy, acne, sunburn, sarcoidosis and angio-oedema, were carefully excluded. Consequently, 63 patients were enrolled who were followed-up for more than 6 months. Clinical data at the first visit for these 63 patients were examined. The patients were treated with Sulfur and Camphor Lotion^®^ [60 mg sulfur and 5 mg Dl-camphor per 1 mL; TSP (Toho Pharmaceutical Co., Ltd, Tokyo, Japan)] or Rozex Gel^®^ [metronidazole 7.5 mg per 1 g; TMP (Maruho Co., Ltd, Osaka, Japan)] until the time analysis. The frequency of topical application was modified according to the presence of irritation. Patients who reported irritation applied the treatment once daily or every other day, while those without irritation applied it twice daily. This adjustment enabled the treatment to be maintained throughout the study. Oral minocycline at 100–200 mg daily was administered in combination for some patients within a total of 28 days from the initiation of the treatment. All patients were advised to avoid common triggers, including sun exposure, skin dryness, significant temperature fluctuations, spicy foods, insufficient sleep and alcoholic beverages.

Among the 63 patients, 49 had their initial visit more than 1 year ago, whereas 14 had their first visit within the past year. Of the 49 patients who first visited us more than 1 year ago, 7 dropped out between 6 and 12 months after their initial visit. The reasons for dropout were as follows: (i) favourable outcomes based on self-assessment (patients #1, #2 and #18); (ii) a change of address (patient #49); and (iii) unknown reasons (patients #13, #22 and #63). The analysis was conducted on a per-protocol basis, defined as including only those patients who completed the scheduled follow-up without major protocol deviations. Therefore, seven patients were excluded from the 1-year clinical course analysis. Consequently, 42 patients were examined 4 times over the course of 1 year. The first examination was performed at the initial visit, which served as the baseline data. Most patients attended their scheduled visits on the designated dates; however, visit dates were adjusted based on individual patient circumstances. Consequently, visits occurring within 39–91 days, 98–232 days and 327–455 days after the initial examination were categorized as the 2-month, 6-month and 12-month visits, respectively.

### Clinical evaluation

The clinical subtypes of rosacea were classified according to the subtype classification by the National Rosacea Society in 2002: (i) erythematotelangiectatic rosacea was defined as facial lesions presenting with flushing and persistent central facial erythema, with or without telangiectasia; (ii) papulopustular rosacea was defined as facial lesions presenting with persistent central facial erythema with transient central facial papules, pustules or both; and (iii) phymatous rosacea was defined as facial lesions presenting with skin thickening and irregular surface nodules.^19^

*Demodex* mite examination was performed as previously described,^20^ and is briefly outlined below. Scales obtained by brushing affected skin regions and/or contents extracted from follicular pustules were treated with potassium hydroxide and examined under a microscope. The result was considered positive if more than one *Demodex* mite was detected.

Patient-reported outcomes were assessed using the Dermatology Life Quality Index (DLQI)^3,21^ and visual analogue scale (VAS) scores for itching, burning sensation, flushing and hypersensitivity. The DLQI is a 10-item questionnaire yielding a score from 0 to 30, with higher scores indicating a greater impact on daily life. Patients recorded a VAS score from 0 to 100 on a 100-mm scale, with a score of 100 representing the worst condition and 0 indicating no symptoms.

Physician-reported outcomes were assessed using the Investigator’s Global Assessment (IGA) scoring system. The IGA score was recorded on a 5-point scale from 0 (clear; no inflammatory lesions and no erythema) to 4 (severe; many small to large papules and pustules or severe erythema).^22^

### Statistical analysis

Statistical analyses were performed using SPSS version 22 software (IBM, Armonk, NY, USA). The linear regression analysis was used to examine the correlation between two quantitative parameters. The Friedman test was conducted to examine changes over time. Multivariate analysis of variance (Manova) was used to assess the differences in change over time for each clinical parameter. The Kruskal–Wallis test was used to evaluate qualitative differences among groups. Values of *P* < 0.05 were considered statistically significant.

## Results

### Results based on baseline data

Data on 63 patients followed up over 6 months, including 42 patients followed up over 1 year, are summarized in Table 1. From the baseline data of the 63 patients, responses to each DLQI question indicated that QOL was frequently impacted by uncomfortable sensations, such as itching, as well as by disfigurement (Figure S1; see Supporting Information). However, social activities, interpersonal relationships, leisure, sports, work and study were less commonly affected by rosacea. The DLQI score indicated that most patients considered rosacea to have small-to-moderate effects on their lives (Table 2).

**Table 1:** Baseline and 1-year follow-up data of the examined patients

	Patients at baseline (*n* = 63)	Patients followed up over 1 year (*n* = 42)
Age (years)		
Range	5–84	5–84
Mean (SD)	40.3 (16.1)	40.9 (16.9)
Sex		
Male	10	5
Female	53	37
*Demodex* mite examination		
Positive	52	31
Negative	11	11
Disease type		
ETR	10	10
PPR	53	32
PR	0	0
Topical treatment option at baseline		
TSP	22	14
TMP	41	28

ETR, erythematotelangiectatic rosacea; PPR, papulopustular rosacea; PR, phymatous rosacea; TMP, Rozex Gel^®^; TSP, Sulfur and Camphor Lotion^®^.

**Table 2:** Distribution of Dermatology Life Quality Index score in baseline

	Effect on patient’s life	Number of patients
0–1	No effect	8
2–5	Small	33
6–10	Moderate	16
11–20	Very large	6
21–30	Extremely large	0

**Figure 1:**
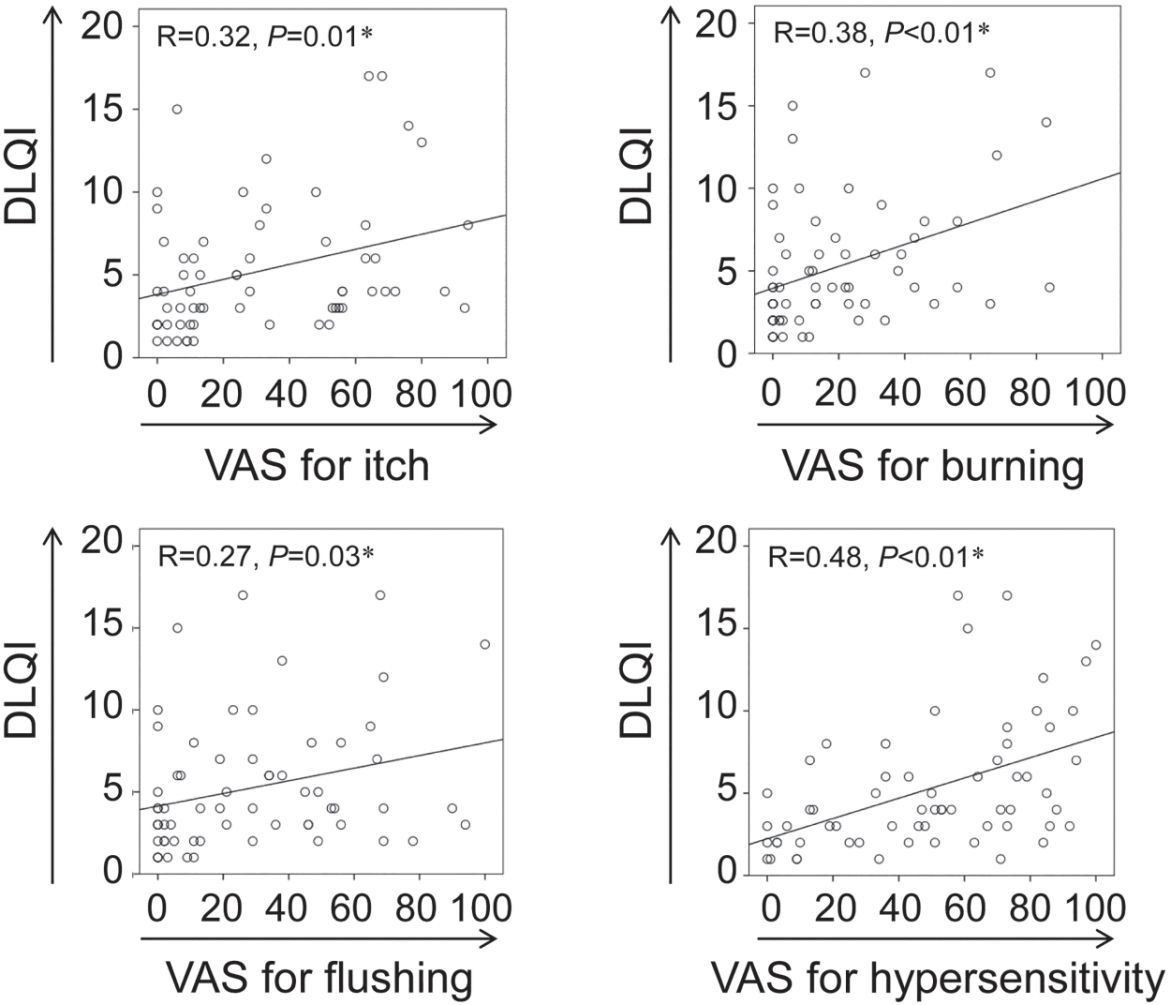
Relationship between Dermatology Life Quality Index (DLQI) score and each visual analogue scale (VAS) score in baseline. The vertical and horizontal axes represent the DLQI score and each VAS score, respectively. The graphs include regression lines. Asterisks (*) indicate significant differences.

The DLQI score was significantly correlated with the VAS scores for itch, burning sensation, flushing and hypersensitivity [correlation coefficients (R) = 0.32, 0.38, 0.27 and 0.48, respectively; *P* = 0.01, *P* < 0.01, *P* = 0.03 and *P* < 0.01, respectively (linear regression analysis)] (Figure 1).

**Figure 2:**
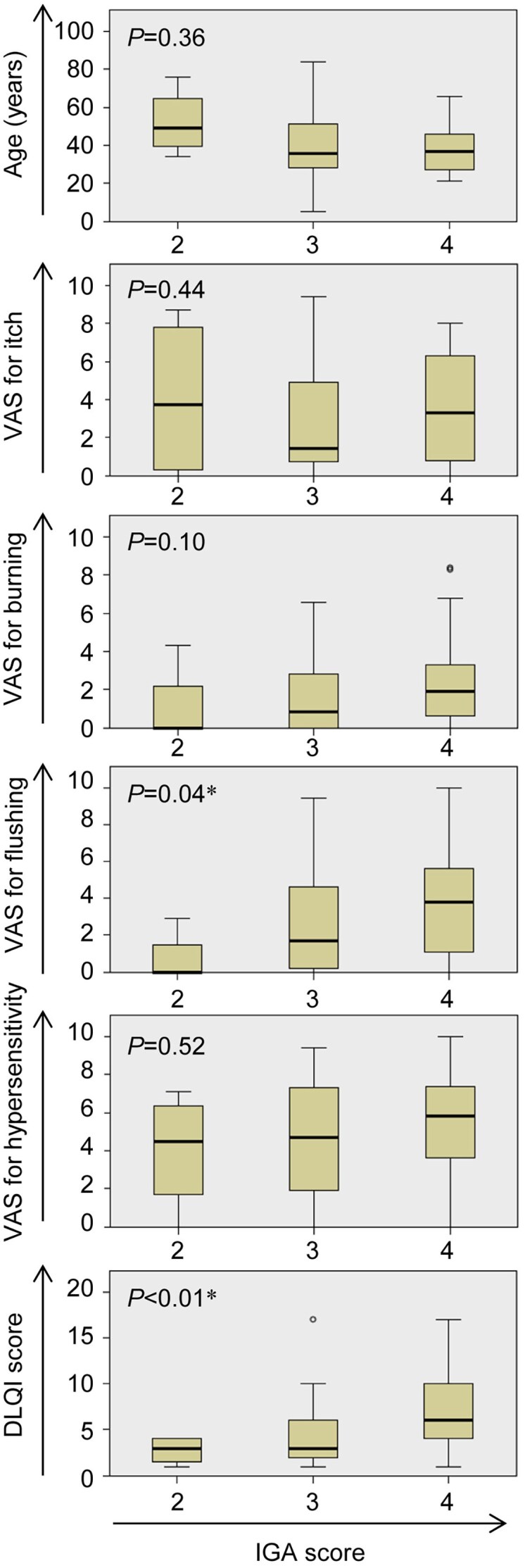
Relationship between Investigator’s Global Assessment (IGA) score and each parameter in baseline. The box chart displays the median, lower and upper quartiles, any outliers, and the minimum and maximum values that are not outliers for each clinical parameter. The vertical and horizontal axes represent each parameter and the IGA score, respectively. Asterisks (*) indicate significant differences. DLQI, Dermatology Life Quality Index.

IGA score was significantly correlated with the VAS score for flushing and the DLQI score (*P* = 0.04 and *P* < 0.01, respectively; Kruskal–Wallis test), while it showed no correlation with age or VAS scores for itch, burning sensation and hypersensitivity (Figure 2).

VAS scores for itch, burning sensation, flushing and hypersensitivity, as well as age and DLQI score, were not significantly different based on sex, results of the *Demodex* mite examination or type of rosacea.

### Results based on clinical course data

**Figure 3:**
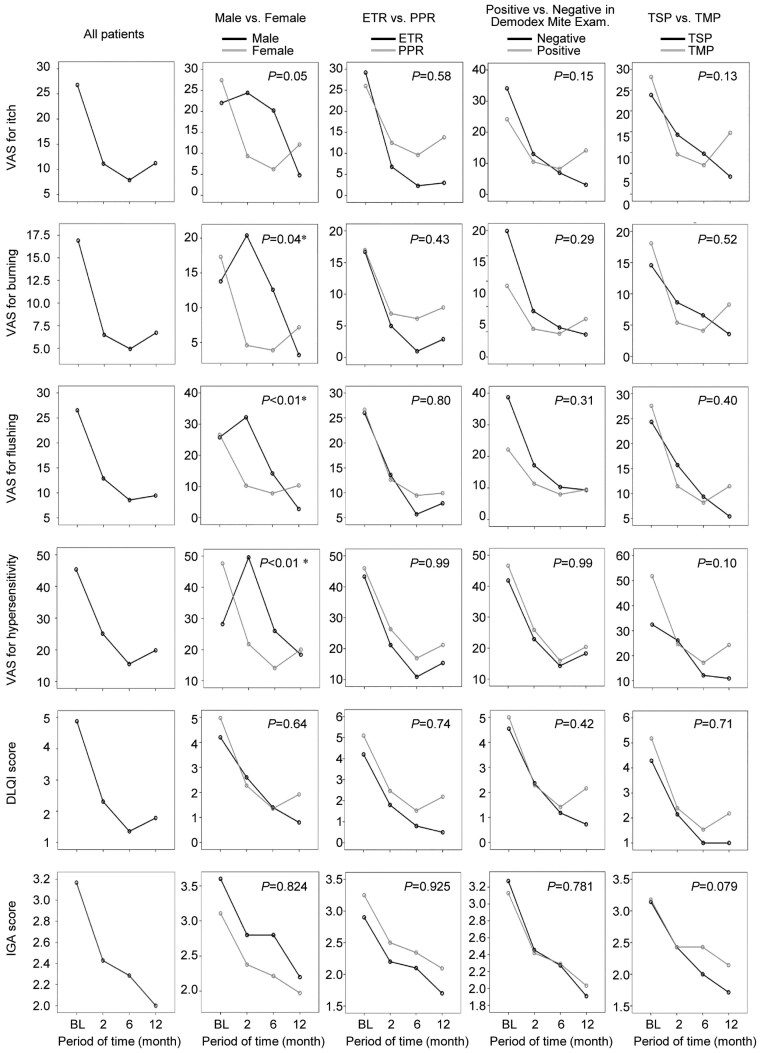
Time course of patient- and physician-reported outcomes across clinical factors. The horizontal axis represents the period of time from baseline (in months). The vertical axis indicates the scores of visual analogue scale (VAS) for itch, VAS for burning sensation, VAS for flushing, VAS for hypersensitivity, Dermatology Life Quality Index (DLQI) score and Investigator’s Global Assessment (IGA) score. Clinical factors include sex, types of rosacea, results of *Demodex* mite examinations and topical therapeutic options. Except for the graphs in the leftmost column, line graphs represented by a black line correspond to men, erythematotelangiectatic rosacea (ETR), negative results for *Demodex* mite examinations and Sulfur and Camphor Lotion® (TSP); line graphs represented by a grey line correspond to women, papulopustular rosacea (PPR), positive results for *Demodex* mite examinations and Rozex Gel® (TMP). Asterisks (*) indicate significant differences.

Data on 42 patients followed up over 1 year are summarized in Table 1. VAS scores for itch, burning sensation, flushing and hypersensitivity, as well as DLQI and IGA scores, were assessed at baseline and at approximately 2, 6 and 12 months thereafter (Figure 3). All scores significantly decreased over time (*P* < 0.01; Friedman test).

Between men and women, there were significant differences in the time courses of VAS scores for burning sensation, flushing and hypersensitivity (*P* = 0.04, *P* < 0.01 and *P* < 0.01, respectively; Manova). However, there were no significant differences in the time courses of the VAS score for itch, DLQI score and IGA score.

Rosacea subtype, results of *Demodex* mite examinations and topical therapeutic options did not affect the trajectories of VAS scores for itch, burning sensation, flushing and hypersensitivity, or those of DLQI and IGA scores.

## Discussion

This retrospective study was conducted at a single institution in a specific geographical area and climate, focusing on a single ethnic population to examine 1-year trends in outcomes following the initiation of treatment in Japanese patients with rosacea. To better reflect the actual clinical status of patients with rosacea in a real-world setting, we conducted a 1-year trend analysis using data from patients who completed all scheduled visits to our clinic over the 1-year period. The 1-year follow-up data initially demonstrated (i) a significant, time-dependent improvement in subjective symptoms affecting QOL and objective findings in Japanese patients with rosacea; and (ii) a sex difference in the clinical course of some subjective symptoms.

The DLQI scores from the baseline results indicate that uncomfortable sensations and disfigurement due to rosacea particularly impair QOL. These results are consistent with several previous reports.^17,23,24^ Notably, the positive correlations between the DLQI total score and VAS scores for itch, burning sensation, flushing and hypersensitivity suggest that uncomfortable sensations directly contribute to an impaired QOL. However, functional ability in daily life related to social activities, interpersonal relationships, leisure, sports, work and study may not be seriously impaired.

In the present study, each VAS score for itch, burning sensation and hypersensitivity was used to identify patients’ complaints arising from excessively activated nociceptive receptors, including transient receptor potential vanilloid 4, transient receptor potential vanilloid 1 and transient receptor potential A1.^1^ These nociceptive receptors are suggested to be involved in the transduction of itch, burning sensations and hypersensitivity, respectively.^1^ Indeed, these parameters showed positive correlations with DLQI scores. The facts suggest the importance of a molecular biological approach to the disease.

Physician-reported outcomes, as measured by the IGA score, were in accord with DLQI scores representing QOL, consistent with previous reports.^25^ However, the IGA score was inconsistent with VAS scores for itch, burning sensation and hypersensitivity. This may indicate that the clinical appearance alone does not represent all aspects of the disease, which includes sensory and appearance-related symptoms. Therefore, diagnostic criteria primarily based on clinical appearance, such as those from the ROSCO panel, may not be suitable for patients – particularly those with darker skin tones – who experience sensory symptoms with minimal visible changes.

Patient-reported outcomes, represented by VAS and DLQI, showed peak improvement at month 6 and a slight rebound at month 12. In contrast, physician-reported outcomes, represented by the IGA, demonstrated continuous improvement over the course of 1 year. These results suggest that the trajectory of patient-reported outcomes might differ from that of physician-reported outcomes over a long period. The definitive reason for this discrepancy is unclear; however, dermatologists should be aware of such differences during long-term follow-up for patients with rosacea.

There were significant differences in the clinical course of burning sensation, flushing and hypersensitivity between men and women. The results suggest that men may not be as accustomed to strictly avoiding triggering factors, such as sunlight and skin dryness, which could contribute to differences in the clinical course between men and women. Nevertheless, because only 5 of the 42 patients who completed the study were men, the results should be interpreted with caution.

There was no significant difference in the 1-year clinical course between patients treated with TSP, a lotion-type agent with a characteristic odour, and those treated with TMP, a cream-type agent. A previous study with an 8-week follow-up also demonstrated no significant difference in outcomes between these topical agents.^20^ In this context, the choice between the two treatments may also depend on individual patient preference over the long term.

The data were obtained from patients at a single institution, all residing in a geographically limited area with uniform climatic conditions and a single ethnic background (Japanese). This relatively homogeneous population offers several advantages in evaluating disease characteristics and interpreting treatment responses. However, given that climatic and ethnic factors are known to influence rosacea phenotypes,^26–28^ these aspects of the study population should be considered when assessing the generalizability of our findings. The study area is located in a humid subtropical climate zone, characterized by high temperatures and humidity in summer, and low temperatures and humidity in winter. This climate type is common throughout much of Japan and southern China. Accordingly, our findings may be applicable to populations of Asian descent living in regions with similar climatic conditions.

A previous Japanese epidemiological report indicated a prevalence of 5.9% for phymatous rosacea.^29^ Although, based on that report, a small number of patients with this subtype might have been expected to be included in our cohort, none was identified. The reason for this discrepancy is unclear; however, it may be attributable to the limited sample size. Nevertheless, the present study reconfirms the rarity of phymatous rosacea among Japanese patients.

A few limitations should be considered. Firstly, the study was conducted retrospectively; therefore, treatment choices were not standardized, and daily lifestyle factors were not strictly controlled. Secondly, the number of patients in the present study was relatively small. Further studies are needed to determine the detailed characteristics of the long-term clinical course of rosacea. Thirdly, iatrogenic rosacea was not specifically identified in the present study, although a previous study in Japanese patients demonstrated seasonal differences in the onset of iatrogenic and noniatrogenic rosacea.^30^ Given that iatrogenic rosacea is frequently encountered in daily clinical practice, this subtype warrants specific characterization in future studies. Fourthly, the data were obtained from patients residing in a limited geographical area. Therefore, the findings should be interpreted with caution.

In conclusion, this study is the first to demonstrate a significant, time-dependent improvement over 1 year in QOL-affecting subjective symptoms and objective findings following the initiation of therapeutic intervention in Japanese patients with rosacea. These findings may inform follow-up care for Japanese patients with rosacea and facilitate exploration of ethnic and geographical variations in the presentation and progression of rosacea.

## Supplementary Material

vzaf124_Supplementary_Data
