# Supplementary figures and images for: One-year outcome trends in Japanese patients with rosacea: insights from a real-world study
Source: Skin Health Dis. 2026 Feb 6;6(2):138–45. doi: 10.1093/skinhd/vzaf124 (PMC13036729; doi:10.1093/skinhd/vzaf124)

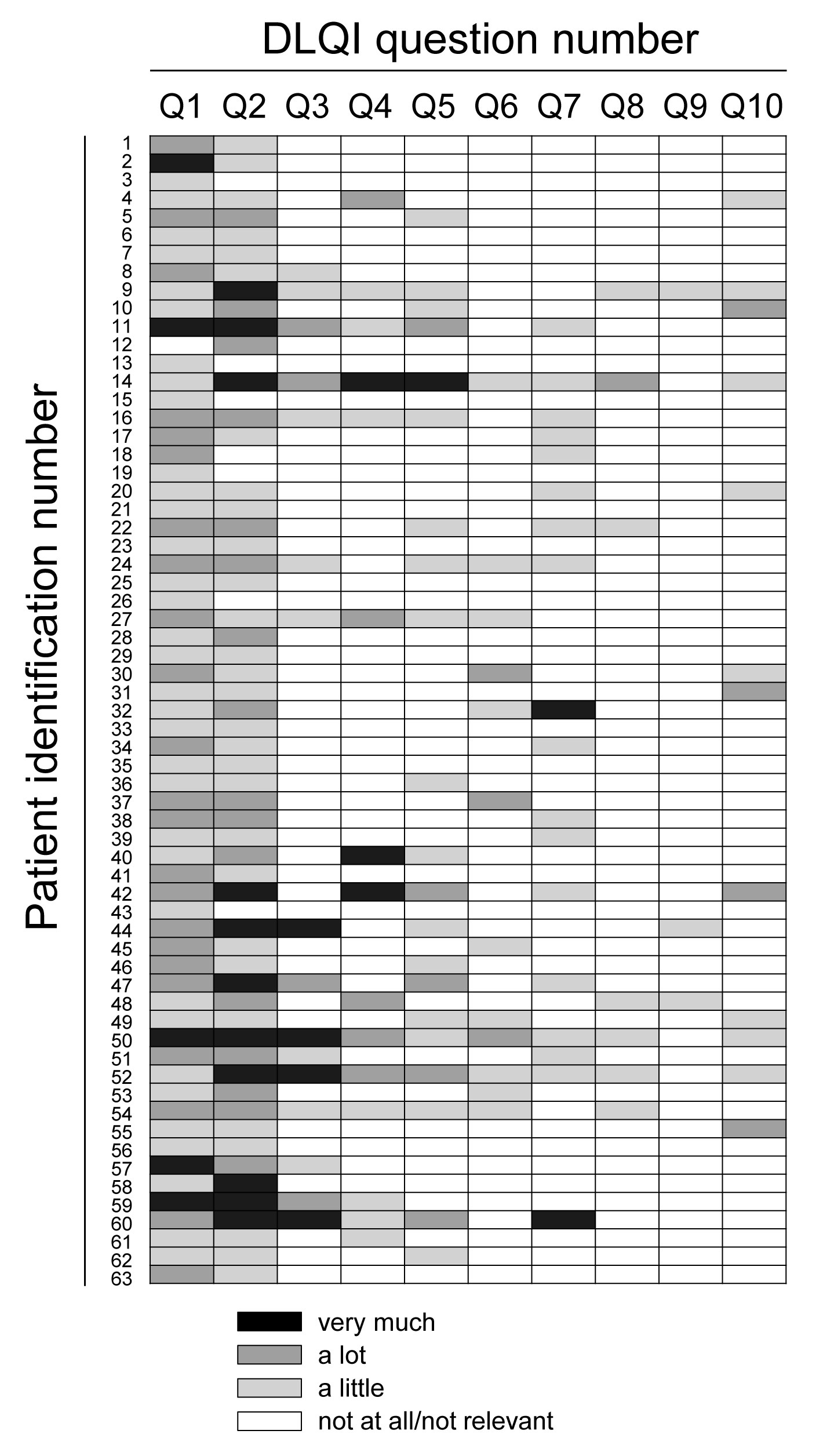

Supplement: vzaf124_Supplementary_Data [file vzaf124_supplementary_data.jpeg]
